# Supplementary material for: Scenarios of Genes-to-Terpenoids Network Led to the Identification of a Novel α/β-Farnesene/β-Ocimene Synthase in Camellia sinensis
Source: Int J Mol Sci. 2020 Jan 19;21(2):655. doi: 10.3390/ijms21020655 (PMC7013532; doi:10.3390/ijms21020655)
Supplement: Supplementary file 1 [file ijms-21-00655-s001.pdf]

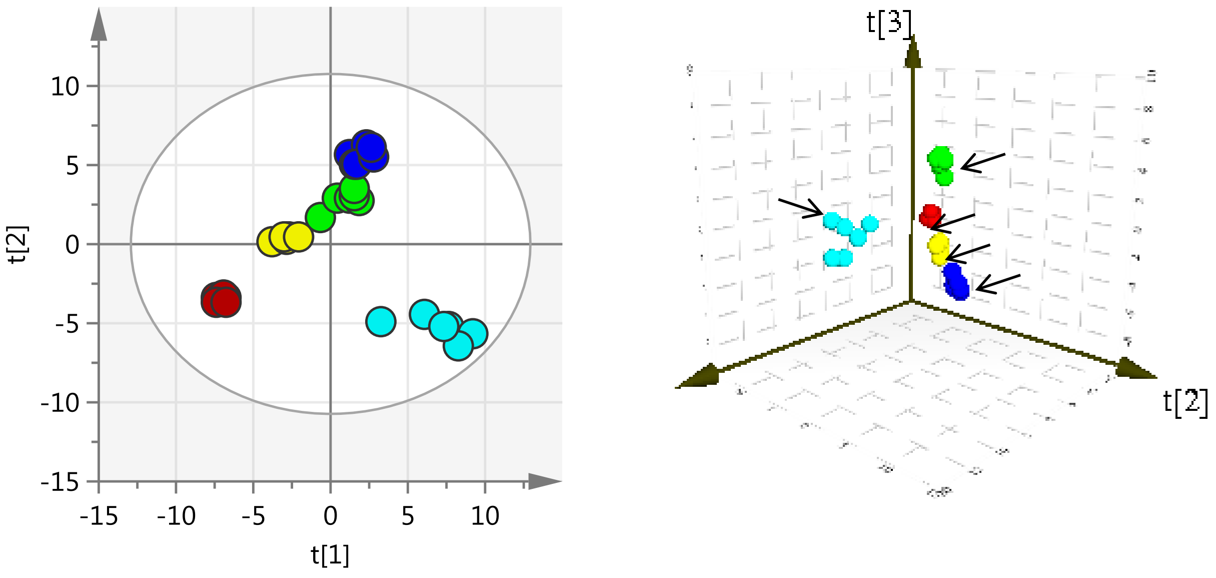

R2X[1]=0.375; R2X[2]=0.258

Supplemental Figure 1. Principle Component Analysis for metabolites from different leaves and stem

Supplemental Table S1: Number of reads get from five tissues of tea plants and quality of transcriptome sequencing

Sample	Number of reads	GC%	Q20%	Q30%
Bud-1	33833529	42	0.999782	0.97464
Bud-2	38754301	43	0.999762	0.974404
FL-1	25833070	42	0.999802	0.966952
FL-2	26044289	42	0.999812	0.968461
ML-1	31705267	47	0.999766	0.965772
ML-2	25758852	44	0.999814	0.968212
SL-1	29076645	42	0.999827	0.968581
SL-2	27800431	42	0.999794	0.96766
Stem-1	30996807	43	0.999819	0.968278
Stem-2	26347796	43	0.999807	0.96791
Total	148075493.5			

Supplemental Table S2: Primers used for q-PCR in this study

Gene	Forward Primer	Reverse Primer
contig17153	CAGGAAACCCACTCTTCCAA	CTTCTTCCCCAAAAACGACA
contig31251	TCCGAGTTCGTGAGTGTCAG	GAAGTCCTTTGCCCCATACC
contig90401	AATCGAGCAGATTTGCAAGG	TTCCCAGAGATGCCGATTAC
contig58508	CCTTCCACTCTTCGTCCTGA	GGTCTCAAGCTCCGCAATTA
contig60663	TGGCCTTTCGAGGTCTATTG	TTCTGTCCATACCGAGCTGTC
contig1798	TTCTAACCTGCCCACTCCAC	GAATCCATGGAAGCCTTTGA
contig3467	TTCCTGACCACCCTCGTTAG	AGCACTTCACCTCCTGATGC
contig133399	TCTTACTCTACACGACGCTCTTCC	TTGGGTTGTTCTTCACAGAGC
contig11606	GACAGGATTGGAGCCGTAAA	AAAGAGCCGAGAGGAGGAAG
contig6307	ATTGTCCGTCTTCCATGCTC	GTGGAGCACCTCAAACATCC
contig45761	GGGATGAGGGAGACCAAAAT	CAAACTCCCTTCCACCTCAA
contig28019	CAACAGAGGAGTCTGCACCA	CTTTAGGGCTTCCCGACATT

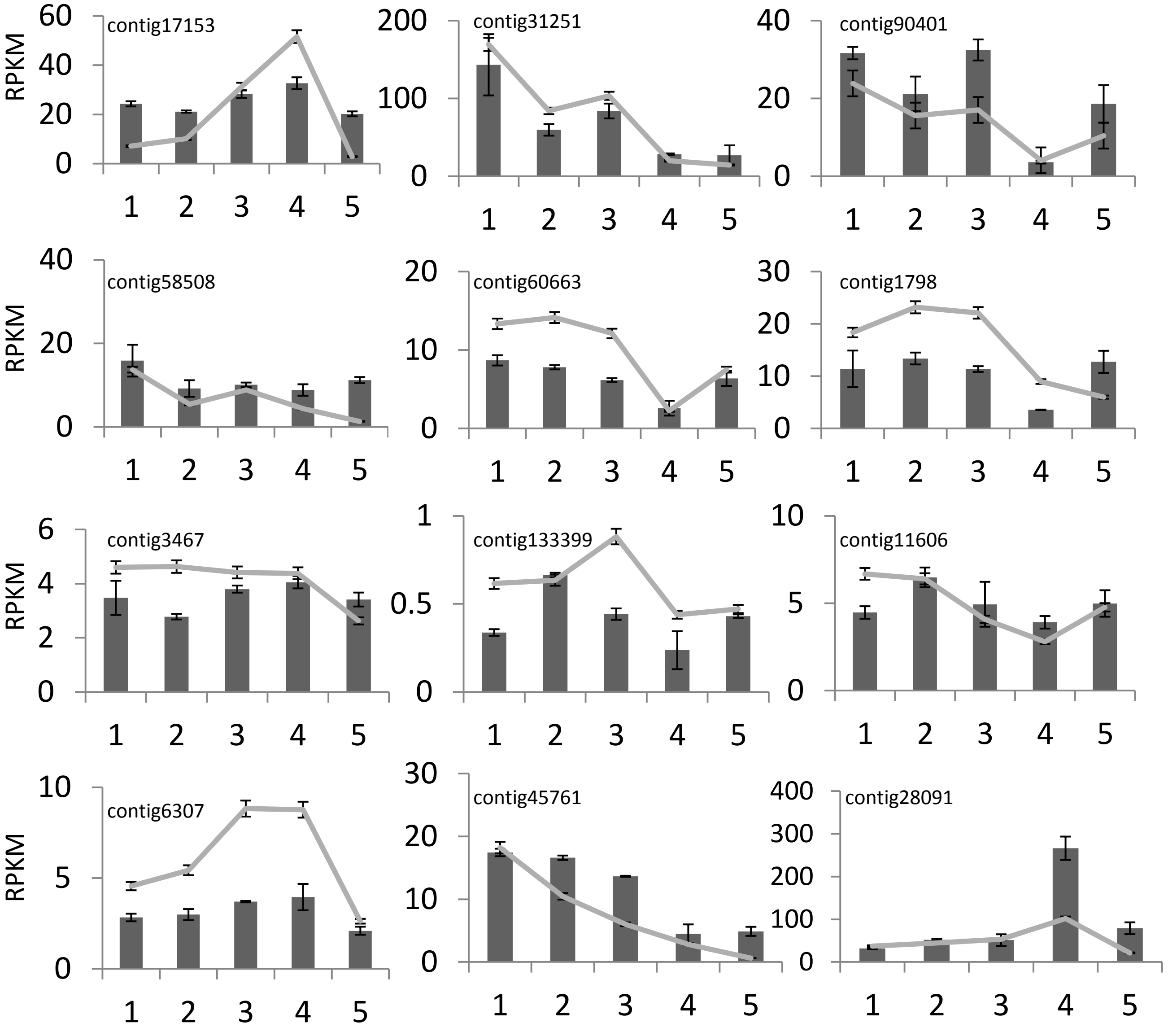

Supplemental Figure 2. Q-PCR results showing that the expression patterns of the 12 genes were in accordance with those of RNA-Seq. 1, 2, 3, 4, 5 means Bud, FL, SL, ML and Stem respectively.
